# Multichannel Approach for Arrayed Waveguide Grating-Based FBG Interrogation Systems

**DOI:** 10.3390/s21186214

**Published:** 2021-09-16

**Authors:** Vincenzo Romano Marrazzo, Francesco Fienga, Michele Riccio, Andrea Irace, Giovanni Breglio

**Affiliations:** 1 Department of Electrical Engineering and Information Technology (DIETI), University of Naples “Federico II”, 80125 Naples, Italy or francesco.fienga@cern.ch (F.F.); michele.riccio@unina.it (M.R.); andrea.irace@unina.it (A.I.); or giovanni.breglio@cern.ch (G.B.); 2 European Organization for Nuclear Research (CERN), 1211 Geneva, Switzerland

**Keywords:** arrayed waveguide grating, fiber Bragg grating, interrogation system, wavelength interrogation, high-frequency optoelectronic system

## Abstract

In this manuscript, an optically passive fiber Bragg grating (FBG) interrogation system able to perform high-frequency measurement is proposed. The idea is mainly based on the use of an arrayed waveguide grating (AWG) device which is used to discriminate the fiber optic sensor (FOS) wavelength encoded response under test in function of its output channels. As made clear by the theoretical model studied in the proposed manuscript, the Bragg wavelength shift can be detected as in linear dependence with the proposed interrogation function which changes with the voltage produced by two (or more) adjacent AWG output channels. To prove the feasibility of the system, some experimental analyses are conducted with a custom electrical module characterized by high-speed and low-noise operational amplifiers. As static measurements, three FBGs with different full width at half maximum (FWHM) have been monitored under wide-range wavelength variation; while, as dynamic measurement, one FBG, glued onto a metal plate, in order to sense the vibration at low and high frequency, was detected. The output signals have been processed by a digital acquisition (DAQ) board and a graphical user interface (GUI). The presented work highlights the characteristics of the proposed idea as competitor among the entire class of interrogation systems currently used. This is because here, the main device, that is the AWG, is passive and reliable, without the need to use modulation signals, or moving parts, that affect the speed of the system. In addition, the innovative multi-channel detection algorithm allows the use of any type of FOS without the need to have a perfectly match of spectra. Moreover, it is also characterized by a high dynamic range without loss of sensitivity.

## 1. Introduction

The progress of technology utilized every day is based on many different physical phenomena that are possible to monitor due to specific sensors. Among a large number of different typologies, in recent decades, research is focusing more and more on fiber optic sensors (FOS) technology; particularly on fiber Bragg grating (FBG) sensors. The FBG sensor is a diffraction grating created by modifying, in a controlled manner, the fiber core in a way to have a periodic perturbation of the refractive index. These sensors work as an optical filter with a reflectance (usually Gaussian) characterized by a central wavelength called Bragg wavelength [1,2,3] which changes with temperature or strain variation. Since they are fabricated with dielectric material, they are characterized by electromagnetic immunity and, together with other advantages, are very suitable in a wide range of environments where other kind of sensors cannot be employed. Moreover, the FBG sensors are very small and is possible to fabricate a large number of them on the same optical fiber. Due to the mentioned advantages, they are employed in many studies regarding the structure health monitoring field (SHM) [4,5,6,7] for vibration monitoring of high buildings or bridges to name but a few. In a more recent work [8], some FBGs were used in the microsurgery field since the material from which they are made is biocompatible and there is no presence of electrical current flowing through the sensor (passive element). The key strength of this sensor is the possibility to manipulate the temperature and strain dependence to many other physical quantities. With appropriate changes on the fiber coating, it is possible to employ the FBG sensor as a humidity sensor [9,10], for radiation monitoring [11] and for cryogenic measurements in superconducting accelerators [12]. It’s worthwile to mention other important works in the field of high-energy physics: where other kinds of sensors may present troubles in invasiveness, reliability and sensitivity, the employment of FBG seems to be the right choice [13,14,15,16]. As in the case study described above, an FBG sensor can be installed in environments with harsh boundary condition and/or hard to reach enviornments for other kind of sensors. It is possible since the interrogation system is installed in a safe place also very far from the sensor section (up to kilometers). The interrogation apparatus, actually, is the true fulcrum of a monitoring system based on FOS technology: the system sensitivity, speed, reliability and the robustness as well, are characteristics of the optoelectronic circuit interrogating one or multiplexed FOS. An interrogation system is, usually, composed by a light source, passive elements for the irradiation of the signal, one or more optical devices for the reflected wavelength discrimination, an electrical section for the acquisition and an algorithm for the manipulation of data. The light source can be a broadband spectrum or a tunable laser. In this latter case, the light source is modulated in time changing in wavelength and the detection algorithm is based on the span time when the wavelength reflection occurs [17]. This kind of interrogation has high accuracy but also a higher cost, with a limit on the speed (best performances on static measurement). Other interrogation systems studied in literature are based on filtering [18], interference [19] or spectral imaging [20].

In several works [21,22,23], many changes in either the optoelectronic section or the detection algorithm have been implemented using, in some cases, an optical filter. This approach poses limitations to the speed and the robustness of the system since the optical filter needs a control signal to be operated. Among the innovations, the arrayed waveguide grating (AWG) seems to substitute well for any kind of active optical filter leading it to have better performance. The AWG is mainly produced for fiber-optic telecommunication since it offers an important Dense Wavelength Division Demultiplexing (DWDM) increasing the communication system capacity. Furthermore, with specific fabrication techniques, is possible to achieve a device completely insensitive to the temperature variation (Athermal AWGs) avoiding the usage of an external temperature controller [24]. It separates a light into its constituent colors as an integrated prism and can be employed as a filter in an interrogation system since it is a robust and reliable device. It detects the FOS central wavelength if contained within its spectrum. Since it is passive, it is possible to increase the operation frequency of the designed system, which will be dependent only on the electronics and the data processing employed, allowing the monitoring of physical phenomena in the range of MHz with a competitive price on the market. In recent years, the AWG-based interrogation system went through a continuous improvement such that many papers were published [25,26,27,28,29,30,31]. The related articles, in a way or another, highlight the disadvantage employing this device: (i) the FOS (the FBG in the most of case studies) has to be positioned exactly in the middle wavelength between two adjacent channels to have the best dynamic range available; (ii) a trade-off between accuracy and AWG channel bandwidth is present; (iii) dynamic range is further limited by non-ideality in the AWG spectrum. Although from a hardware point of view, the solutions seem to be similar to the already cited manuscripts, in the proposed paper, the approach is based on the use of a DSP device opportunely programmed by a novel detection algorithm. The proposed algorithm represents the polynomial nonlinear function between wavelength shift and output of the system. This approach allows us not to use a look-up table method which would require an unsustainable memory usage if the number of AWG output channels grows significantly. Moreover, in the proposed paper, the dynamic range is modular: it is ideally possible to interrogate an FBG employing the whole AWG spectrum without any constrain on the Bragg wavelength position. So, this approach gives a chance to design an AWG with a high number of channels with a low bandwidth value, obtaining an interrogation system with very high accuracy and still having a modular dynamic range of measurement. Finally, since the high-frequency interrogation systems are very expensive on the market, in this work, a high-frequency electronic section was designed ad-hoc and experimentally validated with experimental measurements in the MHz range. This was possible by exploiting the characteristics of the AWG device such as passivity and robustness. This paves the way for a low-cost interrogation system with high resolution, high accuracy and working also for high-speed measurements.

## 2. Theoretical Model

**Figure 1:**
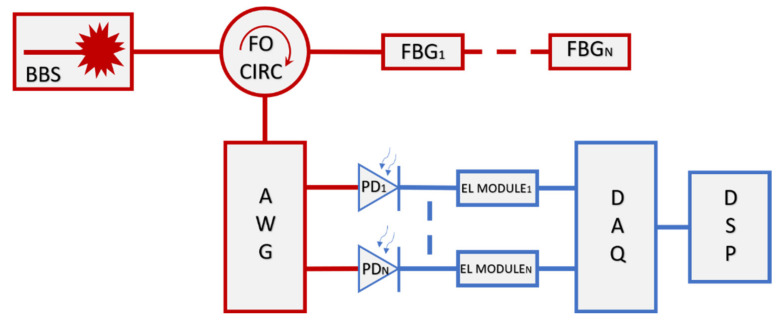
Block schematic about the proposed concept. The red lines describe the optical part, while the blue lines describe the electrical path.

The working principle of the proposed concept depicted in Figure 1 can be described as follows: a broadband source (BBS) containing in its spectrum that of other optical devices, generates a light propagating towards one or more FBG by means of a fiber optic circulator (FO CIRC). Then, the reflected signals are in input of the AWG device. The latter has the task to separate the input into many output channels depending on the correlated central wavelength. Each AWG channel is connected to a photodetector (PD), and then, to an electrical module (EL MODULE). In this way, the optical power is converted in a voltage which is successively acquired by a digital board (DAQ) and processed (DSP) with a detection algorithm.

### 2.1. Analytical Approach

In the case study considered here, the FOS is an apodized FBG (without side lobes) which can be modeled, for its reflectance spectrum, with a Gaussian shape (considering the background noise and other minor effects to be negligible) with order n. The reflectance $B\left(\lambda \right)$ can be expressed as follows:

(1) $$B\left(\lambda \right)=b_{0}\exp{\left[-{\left[{\frac{\left(\lambda -\lambda_{b}\right)}{2c_{b}^{2}}}^{2}\right]}^{n}\right]}^{\;},$$

where $b_{0}$ is the FBG peak reflectance, $\lambda_{b}$ is the central Bragg wavelength while $c_{b}^{2}$ is linked to the full width at half maximum (FWHM, bandwidth) with the following equation:

(2) $$FWHM=2\sqrt{2\ln2}\;c_{b},$$

The AWG transmittance $A_{m}\left(\lambda \right)$ about the generic output channel m can be described with a Gaussian shape of order p  as well and, as the previous case, the background noise can be overlooked:

(3) $$A_{m}\left(\lambda \right)=a_{0m}\exp{\left[-{\left[{\frac{\left(\lambda -\lambda_{am}\right)}{2c_{am}^{2}}}^{2}\right]}^{p}\right]}^{\;},$$

where $a_{0m}$ is the AWG peak transmittance about the generic output channel m, $\lambda_{am}$ is the central wavelength of the same output channel while $c_{am}^{2}$ is linked to the bandwidth as before.

The output voltage produced by the generic m channel can be calculated as the integration in the whole AWG output channel spectrum, taking into account the photodiode responsivity and the electrical module gain. Moreover, the BBS signal can be considered constant in wavelength. This latter consideration can be done since the light source employed has a 3-dB bandwidth much wider than the single AWG channel FWHM. The resulting output voltage in function of the Bragg wavelength variation is:

(4) $$V_{m}\left(\lambda_{b}\right)=a_{0m}b_{0m}\;R\;G\;S\;\int_{\lambda }^{}\exp\left[-{\left[{\frac{\left(\lambda -\lambda_{b}\right)}{2c_{b}^{2}}}^{2}\right]}^{n}\right]\exp\left[-{\left[{\frac{\left(\lambda -\lambda_{am}\right)}{2c_{am}^{2}}}^{2}\right]}^{p}\right]d\lambda^{\;}{,}^{\;}$$

where R is the photodiode responsivity, G is the transimpedance amplifier gain and S is the optical power irradiated by the BBS. The results follow with the first order Gaussian approximation:

(5) $$V_{m}\left(\lambda_{b}\right)=\frac{a_{0m}b_{0m}\;R\;G\;S\;c_{am}b_{0}\sqrt{\pi }}{\sqrt{2}\sqrt{\left(c_{b}^{2}+c_{am}^{2}\right)}}\;exp\left[-{\left[{\frac{\left(\lambda_{b}-\lambda_{am}\right)}{2\left(c_{b}^{2}+c_{am}^{2}\right)}}^{2}\right]}^{\;}\right]=C_{m}exp\left[-{\left[{\frac{\left(\lambda_{b}-\lambda_{am}\right)}{2\Delta_{m}}}^{2}\right]}^{\;}\right],$$

with $\Delta_{m}=c_{b}^{2}+c_{am}^{2}$, related to AWG channel *m* and FBG bandwidth. The output voltage exhibits a clear dependance on $\lambda_{b}$.

### 2.2. Interrogation Function

Considering two adjacent output channels performing voltage $V_{1}$ and $V_{2}$, The interrogation function can be defined as:

(6) $$F_{12}=\frac{V_{1}/C_{1}-V_{2}/C_{2}}{V_{1}/C_{1}+V_{2}/C_{2}}=\frac{exp\left[-{\left[{\frac{\left(\lambda_{b}-\lambda_{a1}\right)}{2\Delta_{1}}}^{2}\right]}^{}\right]-exp\left[-{\left[{\frac{\left(\lambda_{b}-\lambda_{a2}\right)}{2\Delta_{2}}}^{2}\right]}^{}\right]}{exp\left[-{\left[{\frac{\left(\lambda_{b}-\lambda_{a1}\right)}{2\Delta_{1}}}^{2}\right]}^{}\right]+exp\left[-{\left[{\frac{\left(\lambda_{b}-\lambda_{a2}\right)}{2\Delta_{2}}}^{2}\right]}^{}\right]}=\;\tanh\left(\frac{\left[-{\left[{\frac{\left(\lambda_{b}\;-\lambda_{a1}\right)}{2\Delta_{1}}}^{2}\right]}^{\;}\right]-\left[-{\left[{\frac{\left(\lambda_{b}\;-\lambda_{a2}\right)}{2\Delta_{2}}}^{2}\right]}^{\;}\right]\;}{2}\right),$$

which means:

(7) $$F_{12}=\frac{V_{1}/C_{1}-V_{2}/C_{2}}{V_{1}/C_{1}+V_{2}/C_{2}}=\tanh\left(\left[-{\frac{\left(\lambda_{b}-\lambda_{a1}\right)}{\Delta_{1}}}^{2}{}^{\;}+{\frac{\left(\lambda_{b}-\lambda_{a2}\right)}{\Delta_{2}}}^{2}{}^{\;}\right]1/4\right),$$

since ideally the bandwidth of each output channel is equal, $\Delta_{1}$ and $\Delta_{2}$ can be defined as $\Delta_{12}=\left(\Delta_{1}+\Delta_{2}\right)\;/2$ considering a possible mismatch. The equation becomes:

(8) $$F_{12}=\frac{V_{1}C_{2}-V_{2}C_{1}}{V_{1}C_{2}+V_{2}C_{1}}=\tanh\left(\frac{2\lambda_{b}\left(\lambda_{a1}-\lambda_{a2}\right)+\left(\lambda_{a2}^{2}-\lambda_{a1}^{2}\right)}{4\Delta_{12}}\right),\;$$

where can be explicated $\lambda_{b}$:

(9) $$\lambda_{b}=\frac{\mathrm{atanh}\left[F_{12}\right]2\Delta_{12}}{\left(\lambda_{a1}-\lambda_{a2}\right)}+\left(\lambda_{a1}+\lambda_{a2}\right)1/2.$$

If the FBG has a lambda shift in more than two adjacent channels, Equation (9) has to be modified with other values containing information about the adjacent windows (if this is about channel 1–2 then it has to be channel 2–3 and so on). A method to match the Bragg wavelength calculated in the window 1 with the one calculated in window 2 can be given multiplied and dividing for the relative window voltages. The result is a summatory:

(10) $$\lambda_{b}=\overset{\;num\;channels}{\underset{i=1}{\sum }}\left\{\left[\frac{\mathrm{atanh}\left[\frac{V_{i}C_{i+1}-V_{i+1}C_{i}}{V_{i}C_{i+1}+V_{i+1}C_{i}}\right]2\Delta_{i,i+1}}{\left(\lambda_{ai}-\lambda_{ai+1}\right)}+\left(\lambda_{ai}+\lambda_{ai+1}\right)1/2\right]V_{i}V_{i+1}\right\}/\overset{num\;channels}{\underset{i=1}{\sum }}V_{i}V_{i+1}.$$

From this theoretical approach, highlighted by Equation (9), it appears that the interrogated Bragg wavelength is in the form $\lambda_{b}=F\alpha +\beta$, in linear dependance with hyperbolic arctangent of the interrogation function and with a sensitivity given by the slope α which depends on the AWG and FBG bandwidth.

## 3. Methods

In this section, the interrogation system is described with temperature and strain monitoring experimental measurements. The electrical section is described as well. The system was validated by means of quantitative temperature measurements, conducted on an experimental setup composed of three sensors and a heat plate used to control the temperature variation. A calibrated FBG sensor was employed, interrogated with a Micron Optic system and used as temperature reference. The optical signal is acquired at 10 Hz of sampling with an electrical module connected to a 12-bit NI DAQ board controlled by a Graphical User Interface (GUI) at the PC. A strain measurement was conducted as well with a 200 pm bandwidth FBG glued on a metal sheet hitting it with a known weight, sampled at 1 kHz with a frequency analysis annexed. With the same FBG of the previous case, a high-speed measurement was led, sensing the vibration produced by an ultrasound probe in the order of MHz.

### 3.1. Setup Description: Optical Section

**Figure 2:**
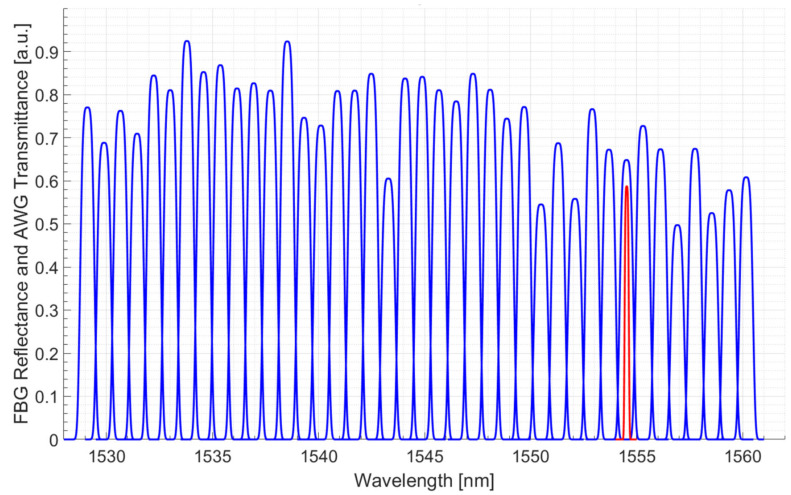
AWG employed spectrum (blue) with the superposition of a 200 pm FWHM FBG sensor (red).

Referring to the set-up scheme depicted in Figure 1, the transmittance spectrum (in blue color) of the used AWG is shown in Figure 2; into the same plot the red curve is the reflectance spectrum of a 200 pm bandwidth FBG as an example. The AWG frequency spacing between two adjacent channels is about 800 pm while the channel bandwidth is about 600 pm (more information about the channels employed are contained in Appendix A). The three FBGs employed during the tests present bandwidths respectively in the order of 200 pm, 315 pm and 500 pm to prove the feasibility of the system for every kind of FBG even with a similar bandwidth between the AWG channel and the sensor.

**Figure 3:**
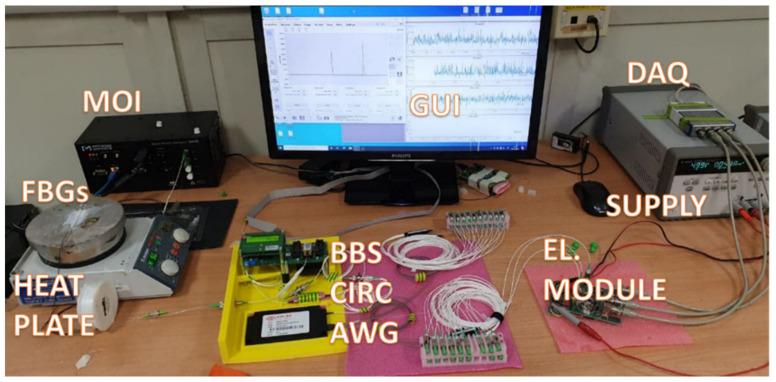
Illustration of the measurement bank with the descripted device.

The optical source is a flat-top SLED with 60 nm of 3-dB bandwidth and a power of 22 mW covering the whole AWG spectrum with a flat trend; the optical circulator is also contained in the source bandwidth, with an insertion loss of 0.6 dB per path uniform in the whole spectrum. The experimental setup is shown in Figure 3.

### 3.2. Electrical Section

**Figure 4:**
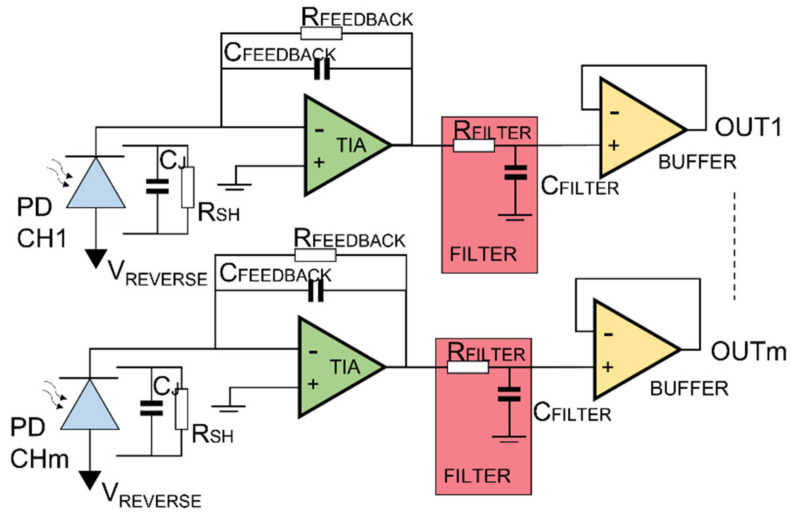
Schematic of the electronic module: representation of a module employing AWG channel from 1 to m to interrogate a single FOS.

**Figure 5:**
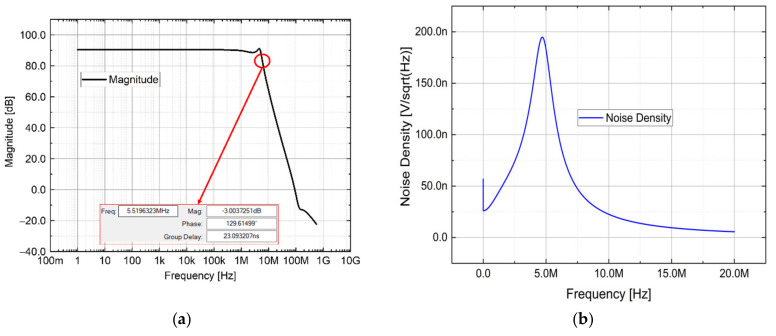
(**a**) Bode diagram and (**b**) noise density simulations of the optoelectronic module.

The circuit scheme of the electrical section is depicted in Figure 4 and is composed of an InGaAs photodetector reverse biased to increase the responsivity at the expense of a greater dark current, a first stage transimpedance amplifier (TIA) with an RC loop on the feedback. The TIA has the aim to convert the photogenerated current in a voltage, then an RC filter and a second buffer stage operational amplifier. The RC feedback loop was dimensioned opportunely to increase the circuit speed without a loss of stability, taking into account the junction capacitance and the operational amplifier input capacitance. The additional pole given by the RC filter downstream is needed to limit the bandwidth, while the buffer is for decoupling. Both transimpedance amplifiers are characterized by low input current, low input capacitance and low noise density. In Figure 5a, the “Bode diagram” is shown with the 3-dB frequency value highlighted, with the aim to show the circuit bandwidth. The latter is about 5.5 MHz with a peak of 5 MHz which is caused by the feedback capacitor. A simulation about the electronic noise is reported in Figure 5b, in which the noise density is in the order of nV/sqrt(Hz), in line with the JFET-based operational amplifier, characterized by very low noise parameters. With the proposed circuitry, since the DAQ board employed uses a 12-bit ADC, the system resolution is in the order of µV, considering the gain of 33 k and the unitary photodiode responsivity, in the order of sub-picometer for the Bragg wavelength calculation.

### 3.3. Algorithm Considerations

**Figure 6:**
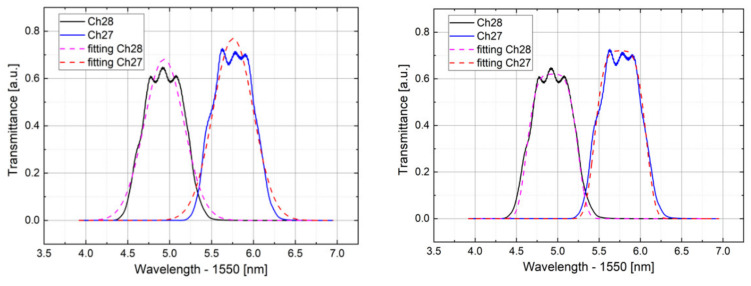
First-order Gaussian fitting of channel 27 and 28 (**left**). Second-order Gaussian fitting (**right**).

The detection algorithm presented in Section 2 is based on some ideal hypotheses which may not be fully satisfied in the actual situation. First of all, for the tests and elaborations, a first-order Gaussian was used to model the single AWG channel transmittance spectrum instead of a second order which, as it is depicted in Figure 6, seems to be closer to real. Theoretically, this may lead to an accuracy decrease with a flat response when the Bragg wavelength is crossing from one window to another.

The conceptual problems mentioned above are related to FBG and characterized with very narrow bandwidth with respect to the AWG channel. From a spectral point of view, it happens because of the FBG reflectance, which is positioned on the channel peak, is so narrow to not integrate the transmittance of the adjacent channel. This consideration involves a lower limit for the bandwidth of FBG that can be interrogated while the upper limit is given by the bandwidth of the AWG channel.

**Figure 7:**
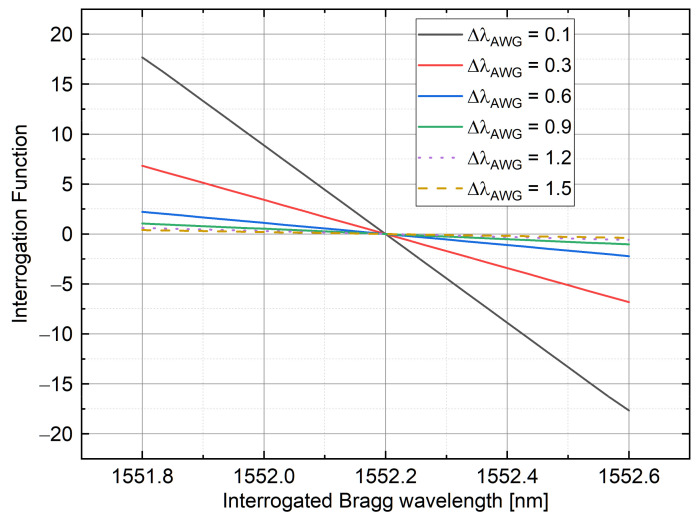
Interrogation function variation versus FBG wavelength for different AWG channel bandwidth values (right).

As said in Section 2, the monitored Bragg wavelength is in the form $\lambda_{b}=F\alpha +\beta$, in a linear relationship with the interrogation function F. Moreover, the sensitivity of the wavelength interrogation is given by the trend of F against λ (shown in Figure 7). The relative slope can be determined through the term α, which depends on the inverse of the term $\Delta_{i,i+1}$ as it is shown in Equation (10). The term $\Delta_{i,i+1}$ is the function of the AWG and the FBG bandwidth since it is directly linked to $\Delta_{ai}^{2}+\Delta_{FBG}^{2}$. This means that by increasing the AWG bandwidth, the dynamic range of interrogation will increase, but the measurement sensitivity will decrease. A trade-off between sensibility and dynamic range is present. This phenomenon is shown in Figure 7 where, through an analytical approach, the $\lambda_{b}$ is changing between 1552.6 and 1553.4 nm, supposing two AWG channels with a frequency spacing of 0.8 nm. 

Concerning the AWG-based interrogation systems, this trade-off leads to a significative decrease of the dynamic range. One of the key strengths of the proposed interrogation system is that the aforementioned trade-off does not affect the wavelength detection: the interrogation function can be extended ideally along the whole AWG spectrum, which means that for a low AWG channel bandwidth value, thus increasing the sensitivity, the dynamic range can be increased employing more channels. The Bragg wavelength is not limited to one window between two adjacent channels, but it can also span among several channels. The detection is performed with an iterative algorithm of the interrogation function avoiding measurement problems related to losses due to connectors, noise due to devices and other parameters.

## 4. Results

In this section, the results obtained from experimental measurements led in static and dynamic variations are reported.

- In order to achieve equivalent static state experimental characterizations, temperature solicitation was sensed by using three FBG having three different FWHM values. The aim of this experimental analysis is to prove that the interrogation capability with the proposed system is not depending on the FBG bandwidth. This peculiarity represents an advantage since, in the most employed interrogation systems based on the reconstruction of the FBG spectrum (e.g., based on tunable laser), a high FBG bandwidth (more than 200 pm) may increase the uncertainty on the Bragg wavelength position. Furthermore, high-bandwidth FBG are less expensive and usually show a Gaussian trend of reflectance closer to the first order.
- As dynamic experiments, the system was tested by using high-frequency strain stresses and an FBG glued on an aluminum plate in order to prove the feasibility. A first vibration test was sensed in the range of hundreds of hertz by beating with a hammer the plate, while, in a subsequent measurement, a pressure signal generated by a piezoelectric probe, in the order to test in MHz range, was sensed as well. The goal of these experiments was to prove that with the proposed optical section, in which passive devices are employed, the proposed concept is able to behave as the currently used interrogation system, also working in applications (as the high-frequency detection) in which the well assessed tunable filter-based interrogators are not suitable due to the limits on the read-out and numerical elaboration speed.

As reported in Section 3.2, the AWG channel spectra are much better modeled by using a second-order Gaussian which, if handled in a different algorithm, gives an equation system without presenting analytical solutions. To overcome this problem, a possible remedy may be given by a look-up-table approach as in [29], although this latter involves onerous data processing, as is explained in Section 4.5. In our experimental analyses, the first-order algorithm explained in Section 2 was employed, approximating both AWG transmittance and FBG reflectance spectrum as first-order Gaussian. To mitigate and overcome the non-ideality error given by the approximation imposed, some corrections were applied:

- A voltage offset given by the electrical circuitry was compensated.
- An additional compensation was done in order to avoid the crosstalk noise among the AWG channels.
- A slope correction was done minimizing the error given by the non-ideal Gaussian trend.

### 4.1. Temperature Measurements

**Figure 8:**
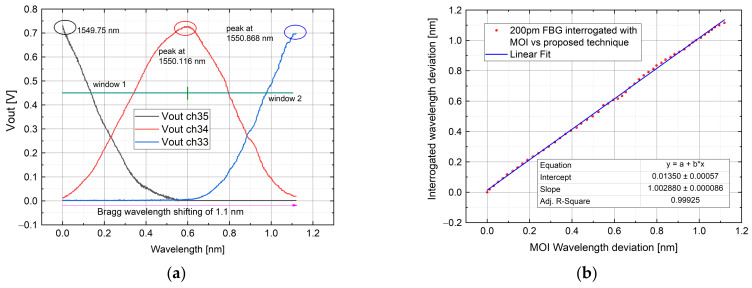
A 200-pm bandwidth FBG sensor was interrogated among AWG channel 35, 34 and 33. The figure shows: channel output voltage (**a**); interrogated wavelength with a commercial system vs. proposed one compared with a linear fit in a dynamic range of measurement of 1.1 nm (**b**). The starting Bragg wavelength position is more than 400 pm over the Ch35 peak.

**Figure 9:**
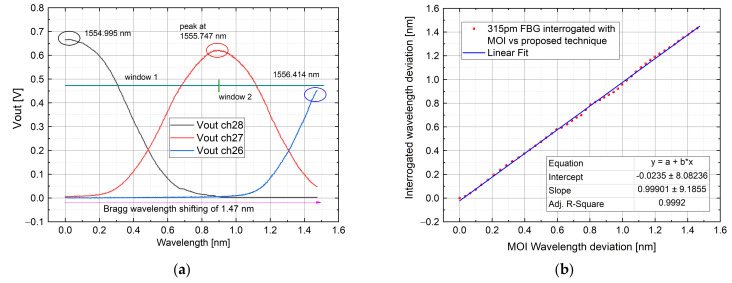
Interrogation of a 315 pm bandwidth FBG among AWG channel 28, 27 and 26: Channel output voltage (**a**); interrogated wavelength with a commercial system vs. proposed one compared with a linear fit in a dynamic range of measurement close to 1.5 nm (**b**). Here, the starting Bragg wavelength position is at the Ch 28 peak.

**Figure 10:**
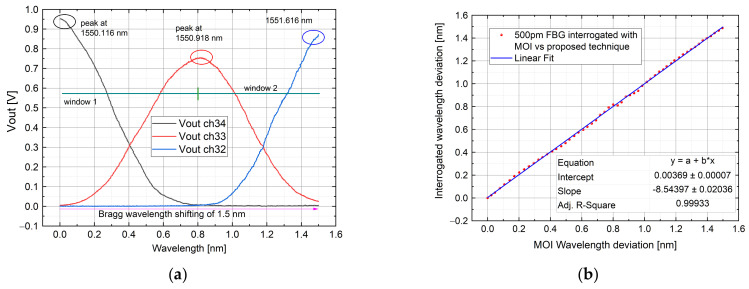
As the last test, the interrogation of a 500 pm bandwidth FBG among AWG channels 34, 33 and 32 is shown with: Channel output voltage (**a**); interrogated wavelength with a commercial system vs. proposed one compared with a linear fit in a dynamic range of 1.5 nm (**b**).

In these kinds of tests, three FBG sensors were interrogated during a temperature variation with the aim to validate the system and ensure the sensing reliability for different FBG bandwidth: 200 pm, 315 pm and 500 pm. The setup used is referred to Figure 3: the FBG sensors were positioned with thermal paste onto a heat plate which is capable to yield the temperature variation, while the voltage signal was acquired by a DAQ board and processed by means of a GUI designed ad-hoc for the test. Each of three Bragg wavelength shifts among 3 AWG channels, for different ranges. In order to verify the correct functionality of the system, each FBG Bragg wavelength was interrogated in the first place with a Micron Optics Interrogator (MOI) as reference, then, successively, with the proposed one with the same temperature shifting (monitored with a calibrated sensor). The agreement between the two approaches is also validated by the linear behavior present in the plot described in Figure 8, Figure 9 and Figure 10. In the next subsections, the abovementioned measurements are reported.

#### 4.1.1. Test by Using 200 pm FWHM FBG

In this test, AWG channels 35, 34 and 33 were employed (the equivalent peak wavelength is 1549.315 nm, 1550.116 nm and 1550.918 nm for each channel). To ensure a linear variation among the interested channels, the initial transient given by the heat plate was avoided. This was done by cooling the FBG in such a way that the Bragg wavelength was positioned before the channel 35 peak, allowing the transient to take place within the window 36–35. The Bragg wavelength was previously interrogated with the MOI system. The validation of the measurement was calculated by comparing the interrogated FBG wavelength shape with the interrogation through the MOI system. The resulting curve (shown in Figure 8) was fitted with a linear equation obtaining an R-square of 0.99925. The measured error ranges from the sub-picometer to less than 20 pm, with a mean error of 2 pm, calculated subtracting point-to-point the two trends. The highest error is in the middle part and is caused by the transition between the windows 35–34 and 34–33. This unwanted alteration is caused by the first-order Gaussian approximation, as described in Section 3.2.

#### 4.1.2. Test by Using 315 pm FWHM FBG

Since the starting Bragg wavelength was changed, for this test AWG channels 28, 27 and 26 were taken into account (each channel peak wavelength is: 1554.940 nm, 1555.747 nm and 1556.555 nm). As before, the FBG wavelength was positioned before the channel 28 peak, to ensure a linear variation among the interested spectrum (ch28–26). The measurement was validated, as in the previous case, comparing the measurement obtained with the proposed interrogator with the one obtained through the MOI system. The comparison results have been linearly fitted and reported in Figure 9. The obtained R-square parameter is equal to 0.9992. The mean error value is 0.2 pm, with a point-to-point error that ranges from the sub-picometer to 20 pm. The highest error is in the middle part of the plot and is due to the transition between windows 28–27 and 27–26.

#### 4.1.3. Test by Using 500 pm FWHM FBG

AWG channels 34, 33 and 32 were employed (the peak wavelength is 1550.116 nm, 1550.918 nm and 1551.721 nm). The FBG wavelength was measured as the previous case, as well as the validation of the measurement. In Figure 10 it is shown the result. As before, the measurement error changes between the sub-picometer value and 20 pm, with a mean error of 0.6 pm. The highest error occurs in the transition between the windows.

The abovementioned trends, as well as the data about the error, are in agreement with the results obtained in literature, as reported in [32]. It’s worthwhile to highlights that, with the proposed multichannel algorithm the dynamic range does not depend on the width of the AWG channel, thus enabling the use of any AWG, regardless of the channel bandwidth. Nevertheless, it must be said that if high sensitivity is needed over a wide measurement range, a high-density AWG (with narrow channel bandwidth) must be employed. In the proposed case studies, the system feasibility for Bragg wavelengths shifting from 1.1 nm to 1.5 nm employing 3 consecutive AWG channels was proved but, if a wider dynamic range is needed, more channels can be monitored and processed. Moreover, the AWG robustness lends itself more than others for high-speed sensing phenomena as was proven in the next sub-paragraphs.

### 4.2. Strain Dynamic Measurements

**Figure 11:**
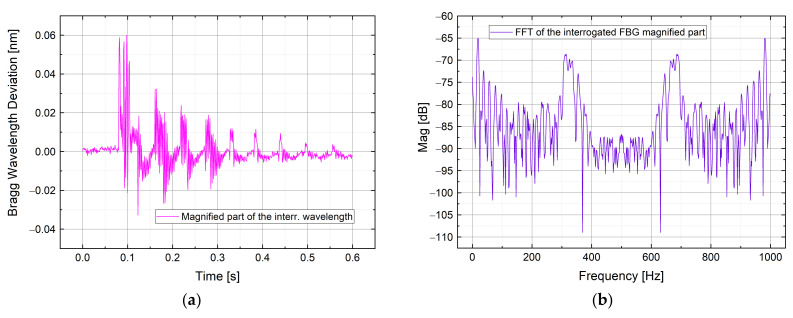
Bragg wavelength deviation interrogated during a strain measurement (**a**), with the relative FFT (**b**).

To test the calibrated algorithm in a non-quasi static lambda variation, a strain measurement in the hundreds of Hz range was done. The AWG channels employed are ch35 and ch33. The FBG was pre-strained in the window 35–34 with the possibility to sense both elongation and compression. The test was conducted by hitting a metal plate of 4 mm thickness where the 200 pm FWHM FBG sensor was glued on. The result is shown in Figure 11 in which is represented the Bragg wavelength variation in time domain (Figure 11a) during the vibration phenomenon, and the corresponding frequency analysis by means of a Fast Fourier Transform (FFT) in Figure 11b.

### 4.3. High-Speed Measurements

**Figure 12:**
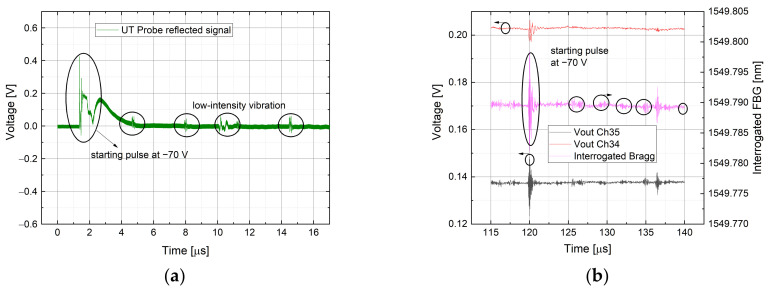

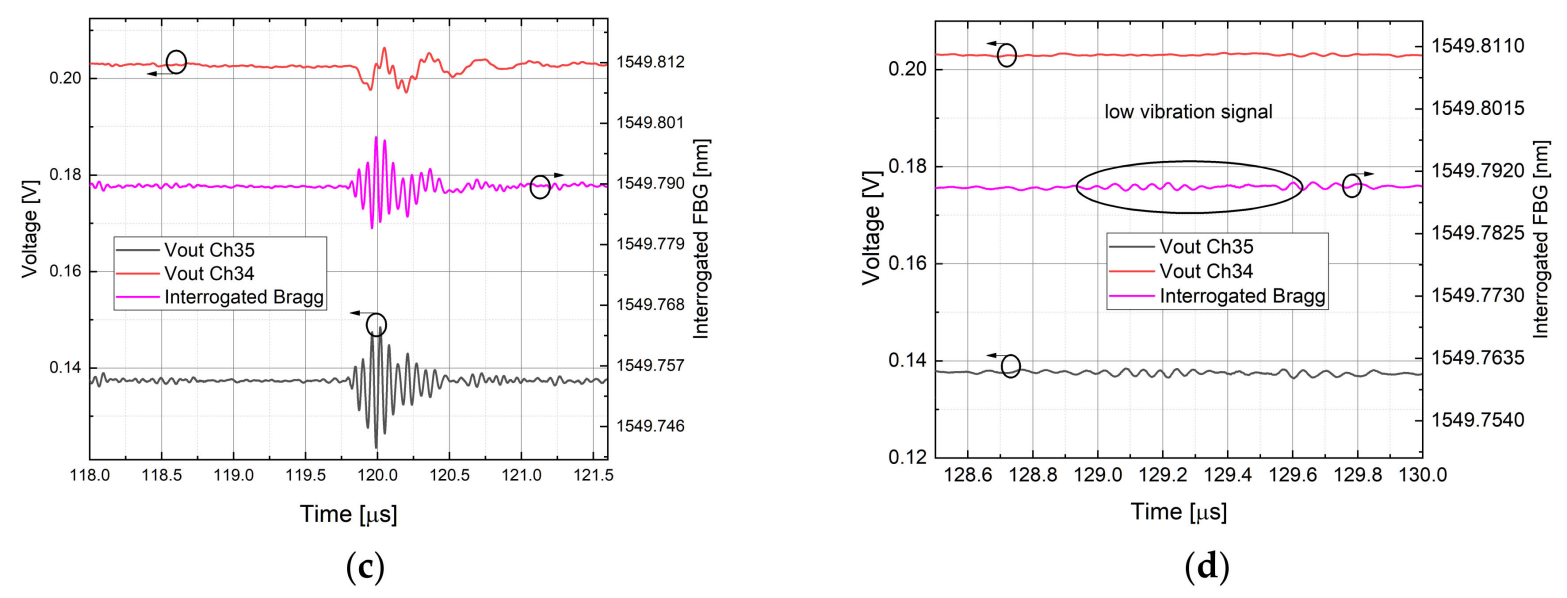
Ultrasound probe reflected signal measurement, with the highlighted part obtained resting the probe on the metal sheet (**a**). Output voltage from the monitoring of channels 35 and 34, with the interrogated FBG in the middle (**b**). Magnified part of the high-speed vibration during the ignition pulse, measured by the FBG sensor (**c**). Magnified about one of the periodic lower intensity vibrations (**d**).

To investigate the capability to sense a phenomenon in the order of MHz, a high-speed measurement was conducted as well. The vibration was generated by an ultrasound probe whose oscillation is in the order of MHz and is sensed through the same FBG glued on a metal sheet employed for the previous case. To check the correct functionality of the probe, the reflected signal was monitored using an oscilloscope and is illustrated in Figure 12a. In this latter, a spike is showed representing the probe ignition with a pulse at −70 V generated by an external circuitry, followed by a series of low-intensity vibrations. In Figure 12b the FBG measurement is depicted: the output voltage of the AWG channel 35 and 34 are represented with the relative interrogated Bragg wavelength obtained with the detection algorithm. It’s worth to note the evolution over time is the same for both electrical and optical behavior, confirming that the vibrations sensed by the FBG are generated via the ultrasound probe. The periodic vibrations with lower intensity are detected as well. In Figure 12a,c magnified part of the probe ignition is represented, while in Figure 12a,d magnified part of the low-intensity vibrations generated by the piezo within the probe is reported.

### 4.4. Relationship Curve between $\lambda_{b}$ and Interrogation Function (F)

**Figure 13:**
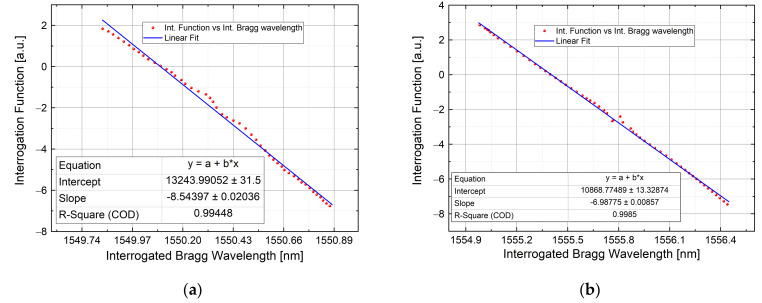

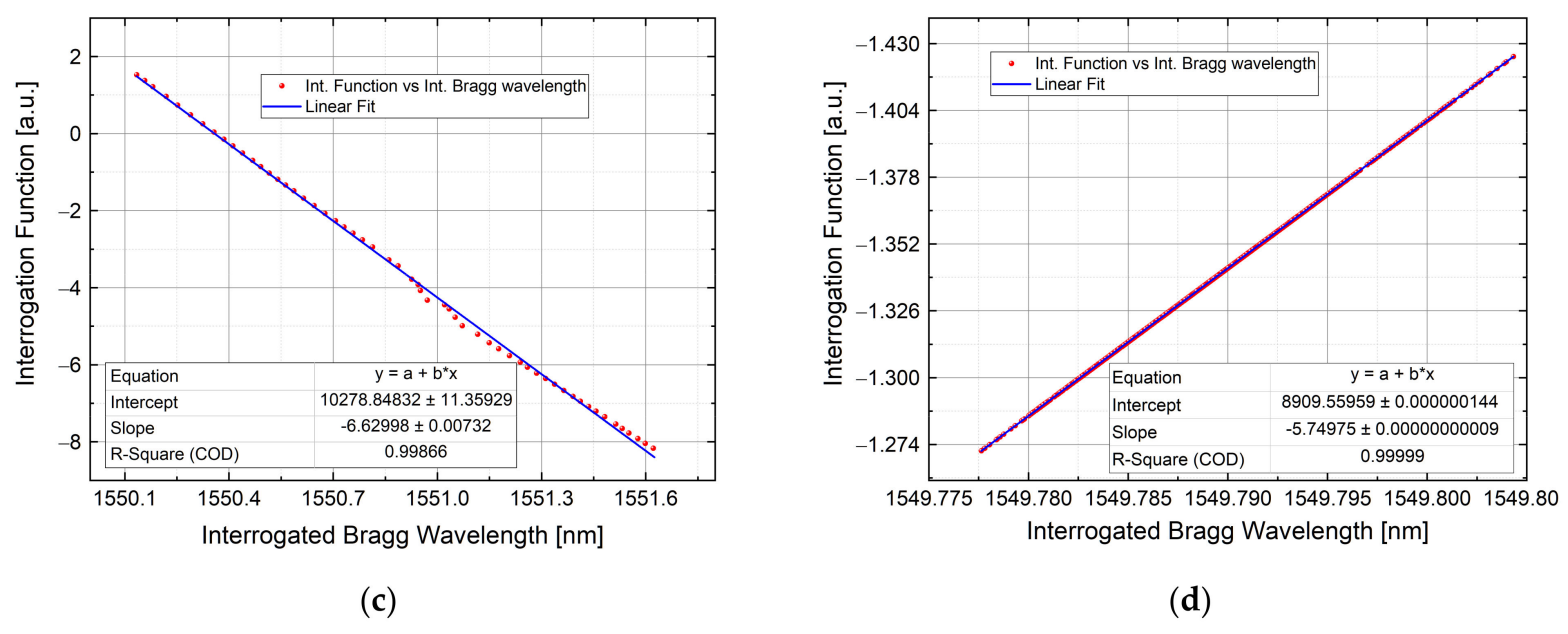
Interrogated Bragg wavelength versus Interrogation Function about 200 pm FWHM FBG during static measurement described in Section 4.1.1 (**a**); about 315 pm FWHM FBG during static measurement described in Section 4.1.2 (**b**); about 500 pm FWHM FBG during static measurement described in Section 4.1.3 (**c**); about 200 pm FWHM FBG during high-speed measurement described in Section 4.3 (**d**).

As demonstrated in Section 2, the monitored Bragg wavelength has a linear behavior with the interrogation function F. The linear trend, given by the equation $\lambda_{b}=F\alpha +\beta$, is also plotted in Figure 7 with theoretical data. To further validate the detection algorithm, in Figure 13 is shown the relationship curve between the Bragg wavelength and F for the static tests as well as the high-speed one. Each curve is superimposed with a linear fit to get the values of α and β, reported in the following Table 1.

### 4.5. Memory Requirement Comparison between Proposed Detection Algorithm and LUT Approach

Considering the generic output voltage between two adjacent channels with a mean excursion of 1 V. The employed DAQ has a resolution of 12-bit (enough to get a resolution in the sub-picometer range), hence, an array of 4096 elements is needed. Since between two adjacent channels, just the half-wave is required, to represent the single AWG output channel 8192 elements are needed. Multiplying by the AWG channels number (40) to have the higher dynamic range possible, 327,680 elements are necessary, with each element represented by 12 bits. Therefore, it is possible to get the final memory requirement by multiplying 327,680 by 12 bits, obtaining almost four Mbits. This result regards only one sensor. Since changing the FBG bandwidth the AWG output changes as well, if three FBGs with different bandwidths are employed (as in the case study) almost 12 Mbits are needed. This result forces the designer to use very high performance and very expensive FPGA.

With the proposed algorithm, information about the AWG channel position and the adopted FBG are included in the equation. Therefore, it is only needed to provide an array of constant numbers (e.g., AWG and FBG channel bandwidth, AWG channel insertion loss, etc.). The main contribution in terms of memory is given by the hyperbolic function that, in principle, can be discretized in a LUT but, at the state of art, many papers have been published with a new algorithm for the implementation of hyperbolic functions in FPGA. For instance, the paper titled “A Configurable FPGA Implementation of the Tanh Function Using DCT Interpolation” [33] reports an architecture in which the hyperbolic tangent is approximated with a very low error, requiring only 1.45 kbits. The hyperbolic tangent is needed for the interrogation of the single FBG. In conclusion, the 1.45 kbits required for the proposed detection algorithm can be compared with the 4 Mbits required for the LUT approach. A difference of 3 orders of magnitude exists.

## 5. Conclusions

In this manuscript, the capability to interrogate one or many fiber optic sensors with a system having a modular dynamic range has been investigated through a multichannel approach based on an AWG device. Starting from a theoretical study, an analytical model of the system has been obtained. It has been demonstrated that the classical 2 channels sensing approach can be overcome by the multichannel methodology without affecting sensitivity of the system but increasing dynamic range. In this work, massive experimental analyses have been conducted with a 40-channels AWG with 800 pm of frequency spacing and 600 pm of channel bandwidth with different kinds of FBG sensors. 

A discrepancy between the theoretical model and real characteristic of optical devices has been taken into account and have been mitigated opportunely. Temperature tests have been reported by using three different FBG sensors characterized by 200, 315 and 500 pm of FWHM. All results have proved the feasibility and the reliability of the system. Measurements have been made in large wavelength variations covering 3 AWG channels. Strain measurements have been reported to test the system in dynamic measurement conditions. A 4 mm metal plate, on which the 200 pm FWHM FBG has been glued, has been hit by a hammer, causing mechanical stresses in the range of hundreds of Hz. Moreover, with the same FBG, a high-frequency measurement has been conducted sensing the vibration generated by an ultrasound piezoelectric source. In both situations, the proposed interrogation system has successfully been used to sense the FBG vibration in the 100 Hz and MHz range, detecting the starting vibration pulse and the low-intensity signal of the piezoelectric source. The abovementioned experimental analyses have been acquired with a custom high-speed and low-noise electrical module composed by a reverse-biased photodetector and a double stage transimpedance amplifier and processed by means of a DAQ board and a GUI performing the detection algorithm. The electrical section offers a 3-dB bandwidth of more than 5 MHz and, since the optical section is completely passive without movable parts, this is the only constrain which limits the speed of the interrogation system. Whether needed, it is also possible to play with the bandwidth-noise trade-off to increase the equivalent speed interrogation performance. The comprehensive digital processing can be easily integrated into an FPGA, avoiding the employment of an operating system. With the proposed interrogation system, it is possible to overcome the limits that afflict the state of the art of the AWG-based systems. In conclusion, the proposed optoelectronic circuit and elaboration stage, which exhibits a large modular dynamic range that works up to 5 MHz with multiplexing capability, constitutes a strong competitor in the field of Interrogator System for FBG arrays for both static and dynamic measurements thanks to its stability, robustness and reliability.

## Figures and Tables

**Table 1:** α and β calculation during static and dynamic experiments.

Test	α	β
200 pm static	−8.54 ± 0.02	13244 ± 31
315 pm static	−6.9877 ± 0.0086	10868.77 ± 13.33
500 pm static	−6.6299 ± 0.0073	10278.85 ± 11.36
200 pm high-speed	−5.75 ± 0.93	8909.56 ± 1.44

## Data Availability

Data underlying the results presented in this paper are not publicly available at this time but may be obtained from the authors upon reasonable request.

## Appendix A

Here follows information about the AWG channels employed as optical filter in the solutions described in this manuscript. The AWG is athermal, with 40 channel and was developed by OpLink (MOLEX). In the next table, Table A1 data about central wavelength, maximum insertion loss, ripple and bandwidth (in terms of frequency) are reported.

**Table A1:** Characteristics about AWG channels employed.

Ports	Wavelength [nm]	Max IL [dB]	Ripple [dB]	FWHM [GHz]
35	1549.315	3.78	0.14	79.22
34	1550.116	3.96	0.19	78.04
33	1550.918	3.94	0.14	78.96
32	1551.721	3.89	0.18	77.80
28	1554.960	3.74	0.16	77.54
27	1555.747	3.74	0.18	78.21
26	1556.555	3.80	0.17	77.45
